# Velocity and Acceleration-Based Differential Plyometric Training Effects on Young Basketball Players

**DOI:** 10.5114/jhk/201139

**Published:** 2025-04-30

**Authors:** Jorge Arede, Irem Pinar Sevin, Mark Williams, Kazimierz Mikolajec, Ricardo Birrento, Wolfgang Schöllhorn

**Affiliations:** 1 School of Education, Polytechnic Institute of Viseu, Viseu, Portugal.; 2 Department of Sports, Exercise and Health Sciences, University of Trás-os-Montes and Alto Douro, Vila Real, Portugal.; 3 Research Center in Sports Sciences, Health Sciences and Human Development, CIDESD, Vila Real, Portugal.; 4 Institute of Sport Science, Otto-von-Guericke-Universität Magdeburg, Magdeburg, Germany.; 5 School of Sport, Rehabilitation and Exercise Sciences, University of Essex, Colchester, Essex, United Kingdom.; 6 School of Psychology and Sport Science, Anglia Ruskin University, Cambridge, Cambridgeshire, United Kingdom.; 7 Institute of Sport Sciences, The Jerzy Kukuczka Academy of Physical Education, Katowice, Poland.; 8 Department of Physical Activity and Sport, Faculty of Sport Sciences, Campus of Excellence Mare Nostrum, University of Murcia, Murcia, Spain.; 9 Human Movement and Sports Science, Faculty of Sport Sciences, Campus of Excellence Mare Nostrum, University of Murcia, Murcia, Spain.; 10 Sports Performance Analysis Association, Faculty of Sport Sciences, Campus of Excellence Mare Nostrum, University of Murcia, Murcia, Spain.; 11 Institute of Sport Science, Training and Movement Science, University of Mainz, Mainz, Germany.

**Keywords:** team sports, variation, movement variability, adolescence, bilateral asymmetry

## Abstract

This study aimed to examine the impact of velocity and acceleration-based differential plyometric jump training on physical performance of youth basketball players. Twenty-six trained youth male players (14.5 ± 1.7 years; U14 [n = 14], U16 [n = 5], and U18 [n = 7]) were grouped into experimental and control groups. The experimental group completed two sessions per week of velocity-based differential plyometric training for 14 weeks (3 sets x 5 jumps with 20-s intervals of passive recovery between jumps and 2-min breaks between sets). Before each repetition, participants received verbal instruction to perform a different fluctuation. The control group continued regular training. Bilateral and unilateral countermovement jump (CMJ) height, the 20-m sprint test, and the Modified 505 Agility (M505) test were evaluated before and after the intervention. The training program yielded statistically significant improvements in the experimental group's CMJ bilateral jump height. Additionally, moderate improvements in the CMJ_R_ (Countermovement Jump Right Leg) and M505_R_ (Modified 505 Agility Right) tests (BF_10_ > 3 to 10) were observed after the training program (δ ranged from 0.66 to 1.12). The control group demonstrated moderate improvements in the M505_R_ (Modified 505 Agility Right) and M505_L_ (Modified 505 Agility Left) tests (BF_10_ > 3 to 10) (δ = 0.65). Models combining different variables provided the best fit for the data in different physical variables. The results indicate that velocity and acceleration-based differential plyometric training can be a suitable strategy for improving physical performance of youth basketball players.

## Introduction

Basketball requires players to have high levels of physical conditioning, which allows them to use their technical and tactical skills effectively during games (de Souza et al., 2024; Schelling and Torres-Ronda, 2016; Ziv and Lidor, 2016). Key physical traits for basketball players include the ability to sprint and jump repeatedly, as well as maintain strength and balance to meet game demands of the game while resisting fatigue (Schelling and Torres-Ronda, 2016; Sosa et al., 2024; Ziv and Lidor, 2016). Activities such as sprinting and jumping require large impulse magnitudes. Therefore, training to enhance impulse-related physical qualities is critical for sporting success (Schelling and Torres-Ronda, 2016).

Various training approaches have been used to enhance the physical capabilities of athletes (Loturco et al., 2023; Sáez de Villarreal et al., 2024), including basketball players (Simenz et al., 2005), but plyometric jump training (PJT) is particularly prevalent (Barrera-Domínguez et al., 2023). The widespread use of PJT in basketball may be attributed to its high transferability to game scenarios (Gonzalo-Skok et al., 2018). PJT utilizes the stretch-shortening cycle (SSC), where muscle-tendon units stretch eccentrically during loading and subsequently shorten concentrically during the push-off phase (Taube et al., 2012). This training induces several physiological and biomechanical adaptations, such as increased motor unit recruitment and the rate of force development, enhancing performance of explosive muscle actions (Markovic and Mikulic, 2010). Studies have shown that PJT significantly impacts muscular strength, linear and change-of-direction sprint speed, balance, and muscular power among basketball players (Ramirez-Campillo et al., 2022).

The successful implementation of PJT typically relies on movement execution that optimizes the contribution of the stretch-shortening cycle (Chu and Myer, 2013). Practitioners often address technical flaws through feedback, coaching cues, and demonstrations of effective techniques (Chu and Myer, 2013). However, despite the clear benefits of PJT, advancements in motor learning within this context remain underexplored. Integrating PJT with motor learning models could further enhance the physical development of young athletes.

One emerging method in motor learning with promising outcomes is differential learning (DL). Unlike traditional motor learning concepts, such as blocked practice, i.e., a linear pedagogical approach that emphasizes repeated exposures to the same skill with low levels of interference or variability (Kim et al., 2018), DL aims to achieve optimal variability in practice. Variations in DL training include changes in movement geometry, velocity, accelerations, and rhythms (Schöllhorn, 2000). This approach allows athletes to explore various aspects of their dynamic movement patterns and retain the most effective solutions as part of the motor learning process (Schöllhorn, 2000). In contrast to linear pedagogic approaches that encourage a technical ideal determined by the coach (Schöllhorn et al., 2022), DL challenges individuals to perform a diverse range of exercises without repetition, simulating the varied environmental conditions encountered during sports-specific performance (Schöllhorn, 2000).

The DL method offers a long-term and non-linear training perspective, which, within the context of athletic development frameworks (e.g., the long-term athlete development model) (Balyi et al., 2013) and the youth physical development model (Lloyd and Oliver, 2012), could be ideal for youth basketball players. Studies on DL in young athletes demonstrate significant improvements in physical qualities (Arede et al., 2021, 2022). For instance, applying DL to repeated sprint training in young basketball players has enhanced sprint and jumping abilities in both sexes (Arede et al., 2021, 2022). Although less frequently used, DL in plyometric training has shown positive results in other athletic populations. For example, DL plyometric programs in volleyball have reduced bilateral asymmetry (Fuchs et al., 2020b). Additionally, a short-term DL plyometric program for university students increased horizontal jump distance and velocity (Rivera et al., 2024).

However, most studies on DL for young athletes focus on manipulating movement geometry, for example changing the body parts position (such as static two arms up, trunk rotation to the left, hands on hip, etc.). Given that the ability to produce large impulse magnitude is crucial in basketball, there is a need for research to examine the effects of DL variations in movement velocity and acceleration on measures of physical performance. Accordingly, this study aimed to examine the effects of movement velocity-based differential PJT on the physical performance capabilities of youth basketball players.

## Methods

### Participants

Twenty-six trained male youth basketball players (mean age: 14.5 ± 1.7 years; average stature: 1.72 ± 0.15 m; typical body mass: 67.2 ± 21.1 kg; basketball experience: 5.7 ± 2.2 yrs) were recruited for this study. These players were drawn from three distinct age groups within the same basketball club academy: U14 (14 players), U16 (5 players), and U18 (7 players). Throughout the experimental phase, spanning from September to December, participants engaged in training sessions three times per week (each lasting 90 min) and competed in one or two matches per week, typically over weekends. To be eligible for the study, players had to be free of injuries and had to have completed all prescribed training sessions in the two weeks leading up to the initial data collection. Participants who missed a testing session or failed to complete at least 90% of the scheduled plyometric training sessions were excluded from the research. Written and informed consent was obtained from the parents of all participants, with the players providing their assent. Approval for the study was granted by the Ethics Committee of the University of Trás-os-Montes and Alto Douro, Vila Real, Portugal (approval code: 20/2019; approval date: 30 January 2019), following the principles outlined in the Declaration of Helsinki.

### Measures

The testing sessions took place in the familiar setting of an indoor basketball court, the same venue where participants routinely trained during the in-season. To maintain consistency, participants were instructed to abstain from vigorous physical activity for 24 h before each testing session and to fast for at least 2 h beforehand. Both pre- and post-intervention testing sessions began with a standardized warm-up lasting approximately 10 min. This warm-up comprised 3–4 min of moderate intensity running and dynamic stretching, followed by 6–7 min of bodyweight exercises focused on muscle strength and endurance, including bilateral and unilateral squats, as well as front and side isometric bridges. Plyometric exercises, such as unilateral vertical jumps were also incorporated into the warm-up routine. Following the warm-up, participants were given time (approximately 2–5 min) to hydrate and dry sweat before physical performance measures were taken. To ensure consistency throughout the study period, all tests were conducted in a standardized sequence, adhering to the principles outlined by the National Strength and Conditioning Association for testing order (Haff and Triplett, 2016). The specific order of the sequence of physical performance tests utilized was bilateral and unilateral countermovement jumps (CMJs), the modified 505 agility test, and the 10-m sprint test. The testing equipment, measurement protocols, and operators remained constant throughout, with three experienced sports science practitioners overseeing the procedures of both the pre- and post-intervention testing sessions.

#### Anthropometrics

The stature was determined with a commercially portable stadiometer (Tanita BF-522W, Japan), with values rounded to the nearest 0.1 cm. Body mass was approximated utilizing a scale (Tanita BF-522W, Japan), with measurements rounded to the nearest 0.1 kg. All measurements were conducted under the protocols established by the International Society for the Advancement of Kinanthropometry (ISAK) by the same researcher, who possessed an ISAK Level 1 accreditation.

#### Jump Height

To assess jump height as a proxy for impulse, participants executed three unilateral (single-leg) and bilateral countermovement jumps (CMJs) from an upright stance on an infrared contact platform (Optojump, Microgate, Bolzano, Italy). Participants self-selected the depth and speed of flexion of the CMJ following the Bosco protocol (Bosco et al., 1983). The CMJ asymmetry index was calculated using the following formula: ASI = 100/Max Value (right and left)*Min Value (right and left)* − 1 + 100 (Bishop et al., 2018). For subsequent statistical analysis, the highest-performed CMJ was selected from the trials conducted.

#### Change of Direction Performance (Modified 505 Agility Test)

Participants were instructed to sprint to a mark positioned 5 m from the starting line, execute a 180° change of direction (COD) utilizing either the right or left leg to push-off, and return to the starting line, covering a total distance of 10 m (Nimphius et al., 2016). They were required to ensure that their entire foot crossed the line marked on the ground at each turn. The total time for the modified 505 agility test was recorded using photoelectric cells placed at a height of 90 cm and separated by 1.5 m (Witty, Microgate, Bolzano, Italy). Each participant completed two sprints with COD for each side, with a rest period of 2 min between each sprint. Players initiated each trial from a standing staggered position, with their front feet positioned 0.5 m behind the first timing gate. The calculation of the COD asymmetry index (ASI) followed the methodology outlined in previous literature (Bishop et al., 2018) using the same formula that was utilized for the CMJ asymmetry.

#### Sprint Times

Split times for 10-m and 20-m distances were recorded and measured using single beam photocell gates positioned 0.9 m above the ground level (Witty, Microgate, Bolzano, Italy). Each sprint began from a standing position chosen by the participant, positioned 50 cm behind the first photocell gate, which activated a digital timer upon movement. Participants completed two maximal 20-m sprints with 2 min of passive recovery between each sprint. The fastest time achieved for the 10- and 20-m distance was selected for statistical analysis.

### Design and Procedures

A non-blind experimental controlled trial, featuring two consecutive data collection phases, was utilized to address the research aims. To ensure participants' familiarity with the physical tests and plyometric exercises, a 20-min familiarization session was conducted a week before the initial data collection. During this session, participants engaged in unilateral and bilateral CMJs, a 10-m sprint test, and a modified 505 agility test. Following a stochastic approach, the experimental group performed various plyometric exercises such as countermovement jumps, squat jumps, Abalakov jumps, and hops. Following the familiarization protocol, all participants underwent testing for CMJ height, 10-m sprint time, and agility performance one week later. These tests were chosen based on their established validity and reliability in previous studies involving youth athletes, including basketball players (Arede et al., 2021, 2022). Additionally, their high portability and feasibility in team settings, particularly under time constraints, made them suitable for this study. Baseline measures including personal (e.g., age, years of basketball experience, team affiliation) and anthropometric (e.g., body mass, height) data were collected at the outset of this testing session. Subsequently, a 14-week intervention phase commenced, during which participants engaged in a plyometric training program twice weekly (on Mondays and Wednesdays) as part of their warm-up routine before their basketball-specific practice sessions. The control group completed regular basketball practices and matches. Following the intervention period, participants were instructed to resume their regular training routines without the plyometric program. One week after the final plyometric session, physical performance measures were reassessed to evaluate changes from baseline to post-intervention.

Over 14 weeks, a differential plyometric training program was conducted in a sports hall with a parquet floor, and players wore their basketball-specific shoes. Group sessions were overseen by a qualified strength and conditioning coach and comprised three sets of 5 jumps, with 20-s intervals of passive recovery between jumps and 2-min breaks between sets. Before each repetition, participants received verbal instruction to perform a different fluctuation (Table 1) or a combination thereof, selected following the principles of differential learning-based training (Schöllhorn, 2000). No movement variation was repeated more than once within a single training session. In the context of differential learning, athletes with prior experience in diversifying motion geometry variables were advised to focus on differentiating movement velocity (Schöllhorn, 2000). Training progressed incrementally, introducing one distinct jump type per week: vertical jumps (e.g., bilateral countermovement, squat, and Abalakov jumps; unilateral squat and countermovement jumps) in weeks 1–5, horizontal jumps (e.g., broad, squat broad, and double broad jumps, hops, double hops, triple hops, and triple crossover hops) in weeks 6–13, and it concluded with lateral jumps (e.g., lateral hops) in week 14. Throughout the training program, no adverse events were reported.

**Table 1:** Training program variations.

Component	Fluctuations	Component	Fluctuations
Body part	Upper body slightly faster than the lower body	Body joint	Right knee slightly faster than the left knee
	Upper body largely faster than the lower body		Left knee slightly faster than the right knee
	Right side slightly faster than the left side		Right knee largely faster than the left knee
	Left side slightly faster than the right side		Left knee largely faster than the right knee
	Right side largely faster than the left side		Ankles slightly faster than hips
	Left side largely faster than the right side		Ankles largely faster than hips
	Upper body faster than the lower body		Ankles slightly faster than knees
	Right-side lower body slightly faster than the left-side lower body		Ankles largely faster than knees
	Left-side lower body slightly faster than the right-side lower body		Knees slightly faster than hips
	Right-side lower body largely faster than the left-side lower body		Knees largely faster than hips
	Left-side lower body largely faster than the right-side lower body		Knees slightly faster than ankles
Body joint	Right arm slightly faster than the left arm		Knees largely faster than ankles
	Left arm slightly faster than the right arm		Hips slightly faster than knees
	Right arm body largely faster than the left arm		Hips largely faster than knees
	Left arm largely faster than the right arm		Hips slightly faster than ankles
	Right ankle slightly faster than the left ankle		Hips largely faster than ankles
	Left ankle slightly faster than the right ankle	Phase	Eccentric phase slightly faster than the concentric phase
	Right ankle largely faster than the left ankle		Eccentric phase largely faster than the concentric phase
	Left ankle largely faster than the right ankle		Concentric phase slightly faster than the eccentric phase
	Right hip slightly faster than the left hip		Concentric phase largely faster than the eccentric phase
	Left hip slightly faster than the right hip	Multiple jumps	Increasing velocity
	Right hip largely faster than the left hip		Decreasing velocity
	Left hip largely faster than the right hip		Interchangeable velocity

### Statistical Analysis

Descriptive statistics, such as mean ± standard deviation, were generated for each measure. The reliability of test measures was computed using an average-measures two-way random intraclass correlation coefficient (ICC) with absolute agreement, inclusive of 95% confidence intervals (CI), and the coefficient of variation (CV). The ICC was interpreted as poor (<0.5), moderate (0.5–0.74), good (0.75–0.9), or excellent (>0.9) (Koo and Li, 2016). Coefficients of variation were considered acceptable if <10% (Cormack et al., 2008). The raw data sets underwent scrutiny for homogeneity and skewness using the Shapiro-Wilk expanded test. In assessing the effects of the 14-week plyometric training program on physical performance measures, distinct Bayesian paired samples *t*-tests (for normally distributed variables) with a Cauchy distribution prior centered on zero and a scale parameter of 0.707 or Bayesian Wilcoxon tests (for variables not adhering to normal distribution) were employed. The Bayesian factor (BF_10_) was then interpreted regarding evidence categories as previously recommended by Wagenmakers et al. (2018): < $\frac{1}{100}$ = extreme evidence for null hypothesis (H_0_ = no main effects), from $\frac{1}{100}$ to < $\frac{1}{30}$ = very strong evidence for H_0_, from $\frac{1}{30}$ to < $\frac{1}{10}$ = strong evidence for H_0_, from $\frac{1}{10}$ to < $\frac{1}{3}$ = moderate evidence for H_0_, from $\frac{1}{3}$ to < 1 anecdotical evidence for H_0_, from 1 to 3 = anecdotical evidence for alternative hypothesis (H_1_), from > 3 to 10 = moderate evidence for H_1_, from > 10 to 30 = strong evidence for H_1_, from > 30 to 100 = very strong evidence for H_1_, > 100 extreme evidence for H_1_. Only those paired comparisons that showed at least strong evidence for supporting H_1_ (BF_10_ > 10) with a percental error < 10 were considered robust enough to describe significant plyometric training effects. The median and 95% central credible interval of the posterior distribution of the standardized effect size (δ) were determined (i.e., the population version of Cohen’s *d* was also calculated for each paired comparison). The magnitude of the posterior distribution of the standardized effect size was classified as trivial (< 0.2), small (0.2–0.6), moderate (0.6–1.2), large (1.2–2.0), or very large (2.0–4.0) (Batterham and Hopkins, 2006). Bayesian repeated measures ANCOVAs (default r scale prior width = 0.5) with age, training experience, and height as covariables were performed to identify the most favored models. The relative contribution of these variables in different physical variables in youth basketball players has been previously confirmed (Carvalho et al., 2019). Statistical analyses were performed using JASP software version 0.13.01 (Amsterdam, Netherlands) and the Statistical Package for Social Sciences (SPSS, v. 28.0 for Mac; SPSS Inc, Chicago).

## Results

All ICCs were excellent (ICC range = 0.96–0.99), and all the CVs were acceptable (CV range = 1.91–8.92%) (Table 2).

**Table 2:** Reliability data for test variables. Data are presented as values with lower and upper confidence limits.

Test Variables	ICC (95% CL)	CV (%) (95% CL)
CMJ (cm)	0.99 (0.99; 1.00)	2.90 (2.17; 3.64)
0–10 m (s)	0.96 (0.90; 0.98)	2.19 (1.61; 2.77)
10–20m (s)	0.97 (0.94; 0.99)	2.46 (1.58; 3.34)
0–20m (s)	0.98 (0.94; 0.99)	1.91 (1.31; 2.52)
CMJ_R_ (cm)	0.98 (0.96; 0.99)	7.12 (4.58; 9.66)
CMJ_L_ (cm)	0.98 (0.96; 0.99)	8.92 (6.64; 11.19)
M505_R_ (s)	0.97 (0.92; 0.98)	4.03 (2.81; 5.24)
M505_L_ (s)	0.98 (0.95; 0.99)	3.84 (2.76; 4.92)

**Figure 1:**
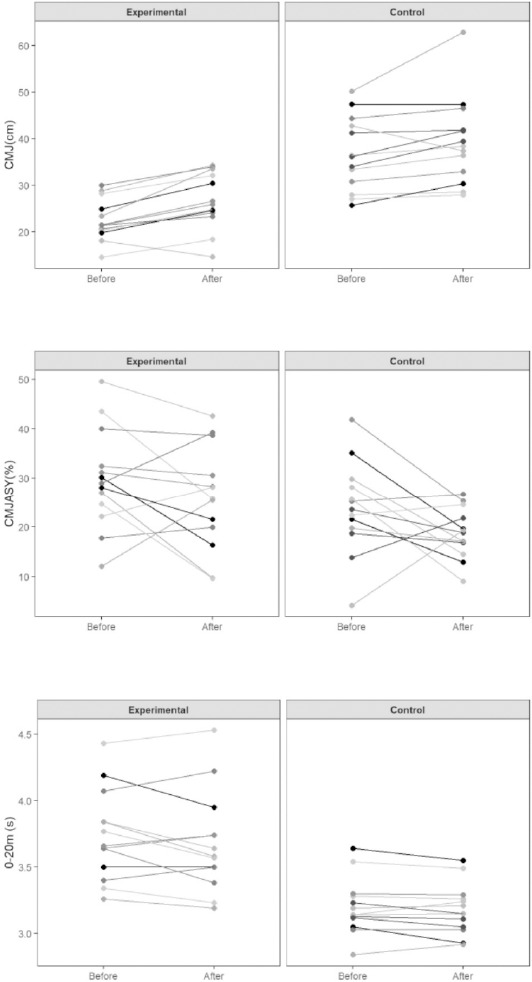
Pre- and post-intervention response comparison for each participant.

After the training program, the experimental group significantly (BF_10_ > 100) improved the CMJ bilateral jump height with a large effect (Table 3, Figure 1). Moreover, in both CMJ_R_ and M505_R,_ moderate evidence for H_1_ (BF_10_ > 3 to 10) was revealed after the training program (δ ranged from 0.66 to 1.12). For the control group, moderate evidence for H_1_ (BF_10_ > 3 to 10) in M505_R_ and M505_L_ (δ = 0.65) was observed.

**Table 3:** Descriptive statistics of the dependent variables and post vs. pre-intervention effects.

Variables		Pretest, Mean ± SD	Postest, Mean ± SD	Differences (95% confidence intervals)	Effect sizes δ (95% confidence intervals)
CMJ (cm)	Experimental	22.53 ± 4.45	26.65 ± 6.06	4.12 (2.34 to 5.91)*****	1.23 (0.48 to 2.04)
	Control	36.96 ± 7.93	39.33 ± 9.41	2.64 (0.14 to 5.13)*	0.54 (−0.01 to 1.13)
CMJ_R_ (cm)	Experimental	12.29 ± 3.89	15.20 ± 4.38	2.92 (1.53 to 4.30)**	1.12 (0.40 to 1.89)
	Control	21.24 ± 5.16	22.87 ± 5.32	1.63 (0.23 to 3.03)*	0.60 (0.03 to 1.21)
CMJ_L_ (cm)	Experimental	12.49 ± 3.25	16.09 ± 5.16	3.61 (1.62 to 5.60)*	0.96 (0.29 to 1.67)
	Control	21.98 ± 5.63	23.55 ± 6.39	1.58 (0.55 to 3.71)	0.37 (−0.14 to 0.92)
CMJ_ASY_ (%)	Experimental	29.81 ± 10.19	25.85 ± 10.50	−3.97 (−10.15 to 2.22)	−0.32 (−0.86 to 0.19)
	Control	23.82 ± 9.36	18.74 ± 5.05	−5.08 (−11.15 to 0.99)*	−0.42 (−0.99 to 0.10)
0–10m (s)	Experimental	2.06 ± 0.16	2.06 ± 0.20	0.00 (−0.05 to 0.06)	0.01 (−0.48 to 0.51)
	Control	1.82 ± 0.10	1.82 ± 0.10	0.00 (−0.01 to 0.03)	0.08 (−0.41 to 0.58)
10–20m (s)	Experimental	1.67 ± 0.21	1.67 ± 0.18	−0.06 (−0.15 to 0.00)*	−0.48 (−1.06 to 0.05)
	Control	1.38 ± 0.12	1.36 ± 0.10	−0.02 (−0.05 to 0.01)	−0.31 (−0.85 to 0.19)
0–20m (s)	Experimental	3.74 ± 0.34	3.67 ± 0.38	−0.06 (−0.15 to 0.03)	−0.33 (−0.87 to 0.18)
	Control	3.20 ± 0.21	3.18 ± 0.19	−0.02 (−0.06 to 0.02)	−0.25 (−0.77 to 0.25)
M505_R_ (s)	Experimental	3.11 ± 0.25	2.95 ± 0.34	−0.16 (−0.29 to −0.04)**	−0.66 (−1.29 to 0.08)
	Control	2.72 ± 0.19	2.63 ± 0.17	−0.09 (−0.15 to −0.02)**	−0.65 (−1.28 to −0.08)
M505_L_ (s)	Experimental	3.08 ± 0.23	2.99 ± 0.27	−0.10 (−0.19 to 0.00)*	−0.52 (−1.10 to 0.03)
	Control	2.74 ± 0.16	2.65 ± 0.14	−0.09 (−0.16 to −0.02)**	−0.65 (−1.27 to −0.07)

Note: * BF_10_ > 1 to 3 (anecdotal evidence for H_1_); ** BF_10_ > 3 to 10 (moderate evidence for H_1_); ***: BF_10_ > 10 to 30 (strong evidence for H_1_); ****: BF_10_ > 30 to 100 (very strong evidence for H_1_): *****: BF_10_ > 100 (extreme evidence for H_1_)

**Figure 2:**
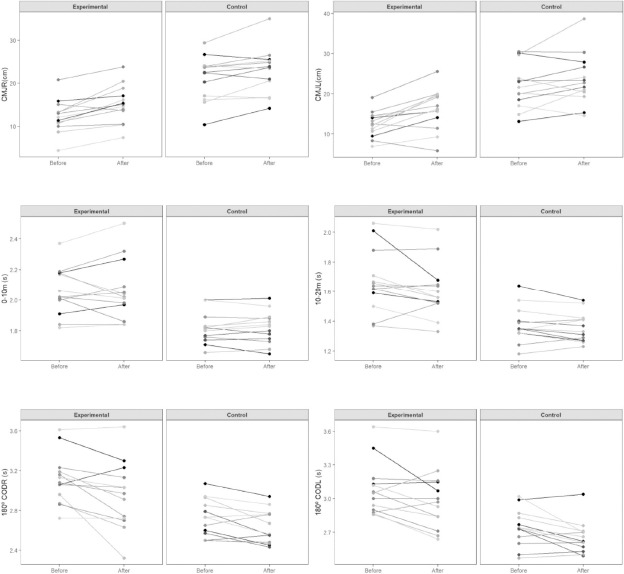
Pre- and post-intervention response comparison for each participant.

According to the Bayesian Repeated Measures ANCOVA, the model including Time + Group + Experience + Age provided the best fit for the data in the CMJ and the CMJ_R_. Regarding the CMJ, strong evidence was observed for the proposed model (BF_M_ = 10.434) over the null model. Considering this variable, the model found was 1.13 times more likely than the second model including Time + Experience + Age. For the CMJ_L_, the model including Time + Experience + Age provided the best fit for the data. There was strong evidence for the proposed model (BF_M_ = 10.809) over the null model. The model found was 1.29 more likely than the second model including Time + Group + Experience + Age. The best model for CMJ_ASY_ was Time + Group, but only with moderate evidence (BF_M_ = 3.543). In the sprinting variables, the model including Group + Height + Age provided the best fit for the data in 0–10-m (BF_M_ = 6.348) and 0–20-m (BF_M_ = 6.420) sprinting time, but only with moderate evidence. For 10–20-m sprinting time, the model including Time + Group + Experience + Height + Age provided the best fit for the data (BF_M_ = 6.008). In the change of direction tests, the model including Time + Group + Height + Age was the best fit for the data on the right (BF_M_ = 10.378) and left (BF_M_ = 8.196) sides. About M505_R,_ strong evidence was found for the proposed model over the null model. Furthermore, this model was 1.538 times more likely than the second model including Time + Height + Age.

## Discussion

This study aimed to examine the effects of movement velocity- and acceleration-based differential PJT on physical performance of youth basketball players. The training program yielded statistically significant improvements in the experimental group's CMJ bilateral jump height. Additionally, moderate improvements were observed in the CMJ_R_ and M505_R_ tests. Meanwhile, the control group demonstrated moderate improvements in the M505_R_ and M505_L_ tests. Overall, models including time, group, experience, and age consistently provided the best fit for various performance variables, with strong evidence in some cases, while group, height, and age were more influential in sprinting and change of direction tests. The interaction between time and group was not included in the best models, suggesting that the changes over time were similar between groups.

Across stages of maturation, CMJ performance has been found to improve in response to training programs (Moran et al., 2018; Ramirez-Campillo et al., 2020a) which are characterized by low training volumes (Ayala et al., 2017; Faude et al., 2013; Williams et al., 2021). Among such programs there are those focused on plyometric-based exercises, as well as others which are broader in content (e.g., jumping, sprinting, and strength-based exercises), i.e., the so-called neuromuscular training programs (e.g., the FIFA 11+). Accordingly, the underpinning mechanisms that contribute to enhanced CMJ performance (e.g., impulse, coordination, and skill) may be considered to require a relatively low training stimulus in youth athletes, especially where exposure to such training may be limited. However, the positive effects of the intervention on CMJ performance in the current study suggest that enhanced performance can also be attained using methods that encourage a high degree of variability in the execution of the programmed exercises. Therefore, even at low training volumes, the implementation of intra-trial variability appears to be an effective method to increase performance. This finding aligns with the observations in a previous study (Hernández et al., 2018), which compared the effects of randomization versus non-randomization of the exercises within a plyometric training program on measures of physical performance in youth basketball players (aged 10.2 ± 1.7 years). Their study revealed that the randomization of plyometric exercises led to significant improvements with moderate to large effects in the CMJ (18%, *d* = 0.60) compared to the non-randomization group (12%, *d* = 0.49). Other studies have also revealed the benefits of training variability on performance outcomes (Kobal et al., 2017; Ramirez-Campillo et al., 2020b) though these have tended to vary the sequencing of training activities rather than variation in how each exercise repetition is performed. Therefore, the findings of the present study extend upon the previous research related to training variation through the novel utilization of differential learning in the execution of each jump repetition.

Owning to the vast array of movement patterns the method encourages, DL has been referred to as an enhanced motor learning strategy that presents each performer with the most appropriate stimulus to benefit their performance (Tassignon et al., 2021). Within the physical preparation of athletes, individualization is understood to be a key principle though, typically, individualized training is applied concerning the choice of the programmed exercises and the external loads that are applied (Barrera- Domínguez et al., 2023; Sarabia et al., 2017). However, whilst individualization of such variables may indeed be of importance, individualized approaches to the athlete’s execution of physical preparation exercises have received limited attention within scientific research. The use of DL involves varying the motor tasks performed, which challenges the neuromuscular system to adapt and refine movement patterns (Horst et al., 2016). Accordingly, the DL approach is understood to foster improvements in neuromuscular qualities and optimize the stretch-shortening cycle (SSC) efficiency, leading to better acceleration and deceleration capabilities during high-intensity actions (Lloyd and Cronin, 2014). Previously, in a study by Fuchs et al. (2020a) differential learning was applied in the execution of the spike action in elite-level volleyball players as part of a modified warm-up across eight sessions. Following the intervention, players were found to have significantly improved performance-related measures for the spike jump, including jump height and approach speed. Within their study, Fuchs et al. (2020a) purposely instructed volleyball players with movement variations that were detrimental to the performance of the spike action before subsequently enabling players to self-determine their optimal variables for the execution of the skill. Although the aims of the current study were different from those of Fuchs et al. (2020a), the similarities in the differential learning methods employed appear to suggest the benefits of such an approach related to the individual performer being able to self-discover their optimum execution strategy. However, in contrast to the study by Fuchs et al. (2020a) whose participants performed variations of an already learned skill, within the current study, young participants performed fluctuated variations of different plyometric-based jumps that were novel. Therefore, it is unlikely that participants in the current study were able to determine the optimal variables to execute each jump variation that was included in the intervention. Instead, the differential learning method in the current study appears to have presented participants with an appropriate stimulus to elicit a change in their CMJ performance.

Given the low volume of the training intervention in the present study, to some extent, the results may be explained by the possibility of a potentiation effect that benefited players in their subsequent basketball practice. By itself, basketball has been found to increase physical capabilities that include CMJ performance (Arslan et al., 2022) which is somewhat highlighted by the improvements observed in the current study’s control group. In the PJT group, the execution of the plyometric intervention within the warm-up may well have augmented their basketball-specific actions which may have contributed to a larger stimulus to enhance explosive performance capabilities. Indeed, the acute effects of plyometric activities on subsequent performance have been previously observed in youth volleyball players (Hammami et al., 2022). Therefore, it is entirely plausible that the findings of the current study may relate to an effect of the so-called post-activation potentiation that occurred in response to the plyometric intervention.

Velocity-based differential PJT appears to be crucial to developing both unilateral and bilateral jumping ability. Movements in team sports often require athletes to produce force bilaterally and unilaterally in variable contexts. The enhancement in that functional capacity allows for better linear sprinting, COD, and jumping performance, which are pertinent to the specific movement demands of basketball (Gonzalo-Skok et al., 2022). Better movement characteristics allow basketball players to be effective in the performance of sport-specific actions (e.g., blocking, rebounding, stealing, passing, and shooting) (Ziv and Lidor, 2016). Therefore, the observed jumping improvements might be related to changes in levels of neuromuscular activation and motor coordination response to specific plyometric training (Gonzalo-Skok et al., 2018). Moreover, combined differential learning and plyometric training can lead to increased joint stiffness, enhanced elastic energy recoil, and the desensitization of the Golgi tendon organ, permitting greater stretching of the elastic component of the muscle (Lloyd and Cronin, 2014). Furthermore, the training-induced adaptations in sensory receptors, such as muscle spindles and Golgi tendon organs, play a crucial role in enhancing reflex control and proprioceptive feedback mechanisms (Radnor et al., 2018). This heightened sensory feedback sensitivity contributes to more precise motor responses and improved movement coordination, particularly in tasks requiring rapid changes of direction and acceleration. However, it is important to acknowledge the dual outcomes of combining plyometric and differential learning training modalities, which may simultaneously increase joint stiffness and optimize elastic energy utilization while potentially desensitizing certain proprioceptive receptors like the Golgi tendon organs (Lloyd and Cronin, 2014). Overall, these mechanisms collectively support the observed improvements in agility performance among the study participants, highlighting the synergistic effects of targeted training strategies on neuromuscular and sensory-motor adaptations.

This combination can consequently contribute to positive performance adaptations, especially in the context of dynamic movements. Indeed, several studies have suggested that differential training may enhance bilateral and unilateral jumping abilities (Arede et al., 2021, 2022). For example, after a nine-week differential repeated sprinting training program, participants displayed higher values of unilateral vertical jumping (except for CMJ_L_ in pre-PHV) compared to pre-test values, irrespective of maturity status (Arede et al., 2022). Similar benefits for unilateral vertical jumping were observed in a pilot study by Arede et al. (2021). Improvements in neuromuscular qualities can be achieved through strategies such as incorporating movement variability and overload, and assisting musculature of hip and knee regions involved in the SSC may be beneficial (e.g., higher peak activity of knee stabilizers muscles or considered concentric peak vertical power/body weight (Meylan et al., 2010)) to have higher unilateral jumping height in youth athletes. Considering another study, a combined jumping directions and force application (vertical and bilateral vs. horizontal and unilateral) plyometric training program led to substantial performance improvements in CMJ_L_ and CMJ_R_ tests (Gonzalo-Skok et al., 2018). After an eccentric-overload training program (EOT) including variable unilateral horizontal movements (VUH) or unilateral lateral movements (VUL), substantial improvements were found in the CMJ_L_ in horizontal jumping and in the CMJ_R_ in vertical jumping (Gonzalo-Skok et al., 2022). However, previous studies in other sports have also shown no improvements in vertical jump performance after horizontal or vertical-orientation training when different contraction speed was used in training (Gorostiaga et al., 2004). Between-studies differences might be attributed to differences in the length of the training intervention, different training loads and volumes used in the discussed studies, the specificity of training (unilateral and bilateral), the speed of movement, and resistance value.

One of the notable findings of the current study was moderate evidence for H1 (BF_10_ > 3 to 10) in the CMJ_R_ observed after a training intervention completed by the experimental group (δ ranged from 0.66 to 1.12). Similarly, moderate improvements in M505R were observed in the experimental group. However, the greater effect of the applied training program on the right leg was unlikely caused by an asymmetry in training loads for the left and right sides. Instead, it is more likely that the left leg was weaker for most participants, which resulted in smaller adaptive changes. Nonetheless, the improvements found in this study surpass those reported in previous research involving youth basketball players, for the same variable (Gonzalo-Skok et al., 2022). To explain this, the current study utilized a method incorporating exercises across various force vectors, whilst the study by Gonzalo-Skok et al. (2022) focused on horizontal force production, which represented lower specificity with the test. Furthermore, other underlying mechanisms should be considered. Firstly, the significant enhancement in agility performance is likely attributable to the training-induced improvements in force production capabilities achieved through plyometric training methods (Jeffreys, 2014). Plyometric exercises enhance the neuromuscular system's ability to recruit motor units efficiently, increase firing frequencies, and improve muscle force and the rate of force development in the lower limbs (Lloyd and Cronin, 2014). These adaptations are crucial for enhancing performance in activities requiring rapid changes of direction, such as the M505 test. Despite this, previous studies have shown that different training programs can significantly improve bilateral asymmetries in youth basketball players (Arede et al., 2021, 2022). However, although various jump types, including unilateral movements, were introduced, the focus on differential learning and movement variation did not specifically intend to address existing imbalances. Indeed, a previous study that targeted single-leg countermovement jump bilateral asymmetry applied traditional training methods (Bettariga et al., 2022). Accordingly, the DL method utilized in the program of the current study may have been insufficient to induce significant changes in bilateral symmetry, especially for those with severe imbalances.

Our findings indicate that a 14-week differential plyometric training program with a focus on vertical movements did not significantly improve 0–10-m sprint times. Elsewhere, plyometric training has been shown to improve 0–10-m sprinting times in youth and adult basketball players, with a significant effect size (Ramirez-Campillo et al., 2022). Additionally, previous interventions of shorter-duration (6–7 weeks) have revealed improvements in pre-pubertal and pubertal players (Gonzalo-Skok et al., 2018; Sáez de Villarreal et al., 2021). Furthermore, our approach was less effective compared to an 8-week combined resistance and plyometric training program among Portuguese pubertal basketball players (Arede et al., 2019), suggesting that either more intense or more specific, or both, training targeting the hip and knee musculature may be needed for better sprint performance (Jaitner and Pfeiffer, 2003). This could be due to the principle of specificity; the varied exercises with dominant vertical orientation may not have targeted the specific muscle groups and neuromuscular patterns crucial for horizontal sprinting. Effective sprinting requires key events in the running cycle, which may not have been adequately addressed by the differential learning plyometric training program. Additionally, the variability in movement patterns could have diluted the specificity needed for optimizing sprint performance. Individual differences in training response also likely contributed to the null effect, emphasizing the need for personalized training programs that align more closely with sprinting demands (Lahti et al., 2020). Future research should focus on tailoring plyometric interventions to improve sprint-specific performance and account for individual variability in response. A possible approach to cope with the problem of transfer between horizontal and vertical jump training and movement directions of the influenced movement was previously suggested (Lahti et al., 2020), implementing constantly changing heights and distances of hurdles during the jumping series. In this way, differential jump training allowed a direct transfer to the jump throw in handball, which is characterized by a slight horizontal movement as well (Lahti et al., 2020).

We present additional evidence supporting the efficacy of a novel plyometric training method that fosters self-organization. Consistent with Fisher's statistical analysis, our findings suggest that further investigation into plyometric training using differential learning holds potential. Additionally, as our analysis primarily adhered to the Fisher’s framework, supplemented by effect size measurements in line with Neyman-Pearson principles (Neyman and Pearson, 1992), we need to refrain from making broad generalizations (Nuzzo, 2014). Nevertheless, we recommend conducting further studies encompassing a range of participants, including recreational athletes, highly trained individuals, elite, and world-class athletes. Longitudinal research, in which the same subjects are assessed over several months, is also advocated to better understand the intricate, ongoing adaptations in individuals.

## Conclusions

The observed improvements suggest that incorporating plyometric exercises into routine training regimens can be particularly beneficial for youth basketball players, improving key physical attributes necessary for basketball performance. The differential learning-based approach employed in the plyometric training protocol, which emphasizes varied and non-repetitive movement patterns, may have contributed to the observed performance gains by promoting neuromuscular adaptation and motor learning. Practitioners working with youth basketball players are encouraged to incorporate differential plyometric training into their routines to enhance athletic performance. Overall, this study adds valuable evidence to the growing body of literature on youth athletic development, highlighting the potential of differential learning-based training programs to enhance critical performance attributes in youth basketball players.
